# MiR-34a-3p alters proliferation and apoptosis of meningioma cells *in vitro* and is directly targeting SMAD4, FRAT1 and BCL2

**DOI:** 10.18632/aging.101201

**Published:** 2017-03-23

**Authors:** Tamara V. Werner, Martin Hart, Ruth Nickels, Yoo-Jin Kim, Michael D. Menger, Rainer M. Bohle, Andreas Keller, Nicole Ludwig, Eckart Meese

**Affiliations:** ^1^ Institute of Human Genetics, Medical School, Saarland University, 66421 Homburg/Saar, Germany; ^2^ Institute for Clinical and Experimental Surgery, Medical School, Saarland University, 66421 Homburg/Saar, Germany; ^3^ Institute of Pathology, Medical Center, Saarland University, 66421 Homburg/Saar, Germany; ^4^ Clinical Bioinformatics, Saarland University, 66123 Saarbruecken, Germany

**Keywords:** meningioma, miR-34a-3p, SMAD4, FRAT1, BCL2

## Abstract

Micro (mi)RNAs are short, noncoding RNAs and deregulation of miRNAs and their targets are implicated in tumor generation and progression in many cancers. Meningiomas are mostly benign, slow growing tumors of the central nervous system with a small percentage showing a malignant phenotype.

Following *in silico* prediction of potential targets of miR-34a-3p, *SMAD4*, *FRAT1*, and *BCL2* have been confirmed as targets by dual luciferase assays with co-expression of miR-34a-3p and reporter gene constructs containing the respective 3'UTRs. Disruption of the miR-34a-3p binding sites in the 3'UTRs resulted in loss of responsiveness to miR-34a-3p overexpression. In meningioma cells, overexpression of miR-34a-3p resulted in decreased protein levels of SMAD4, FRAT1 and BCL2, while inhibition of miR-34a-3p led to increased levels of these proteins as confirmed by Western blotting. Furthermore, deregulation of miR-34a-3p altered cell proliferation and apoptosis of meningioma cells *in vitro*.

We show that *SMAD4*, *FRAT1* and *BCL2* are direct targets of miR-34a-3p and that deregulation of miR-34a-3p alters proliferation and apoptosis of meningioma cells *in vitro*. As part of their respective signaling pathways, which are known to play a role in meningioma genesis and progression, deregulation of *SMAD4*, *FRAT1* and *BCL2* might contribute to the aberrant activation of these signaling pathways leading to increased proliferation and inhibition of apoptosis in meningiomas.

## INTRODUCTION

With an overall frequency of 35 % meningiomas are one of the most frequent primary tumors of the central nervous system [1]. Meningiomas are classified into three World Health Organization (WHO) grades [2]. About 80 % of all cases are WHO grade I meningiomas [3]. The higher-grade meningiomas with WHO grade II or III account for 18-20 % and 1-2 % of all cases, respectively [3]. The most frequent cytogenetic aberration in benign WHO grade I meningiomas is the partial or complete loss of chromosome 22 that is associated with the loss of the neurofibromatosis 2 (*NF2*) gene [4]. In WHO grade II and III meningiomas additional cytogenetic aberrations such as losses on chromosomes 1p, 6q, 9p, 10, 14q and 18q and gains on chromosomes 1q, 9q, 12q, 15q, 17q and 20q are observed [4]. Recently, somatic protein-altering mutations were found in a subset of meningiomas without *NF2* mutations [5, 6]. The affected proteins include transcription factors like Kruppel like factor 4 (*KLF4*) and components of the Phosphatidylino-sitol-3-kinase (PI3K) signaling pathway like AKT serine/threonine kinase 1 (*AKT1*) and Phosphatidylino-sitol-4,5-bisphosphate 3-kinase catalytic subunit alpha (*PI3KCA*) [5, 6].

Micro (mi)RNAs are about 19-25 nucleotide long, small noncoding RNAs with a pivotal role in the posttranscriptional regulation of gene expression [7]. They bind to specific sequences in the 3' untranslated region (3'UTR) of mRNAs leading either to translation repression or degradation of the target mRNAs [7]. Recent studies have shown that miRNAs play a crucial role in many cancer related cellular signaling pathways involved in regulation of proliferation, invasion, migration, apoptosis and epithelial-mesenchymal transition (EMT) [8].

In meningiomas several signaling pathways have been identified as deregulated including RB/p53, Wnt/β-catenin, transforming growth factor β (TGF-β), mitogen-activated protein kinase (MAPK), Phosphatidylinositol-3-kinase (PI3K)/Akt and vascular endothelial growth factor (VEGF) [9, 10]. A key activator of the Wnt pathway, β-catenin, has been validated as target of miR-200a, which plays a crucial role in meningioma cell proliferation [11]. For the RB/p53 pathway, retinoblastoma 1 (Rb1), a crucial factor at the G1/S-phase checkpoint in cell cycle progression, has been validated as target of miR-335 [12]. Overexpression of miR-335 led to a significant increase of cell proliferation and inhibition of cell cycle arrest in the G0/G1 phase whereas inhibition of miR-335 had the opposite effects in human meningioma cells [12].

In our previous study we identified miRNAs deregulated in meningiomas of different WHO grades and histological subtypes, including miR-136, −34a-3p, −376c, −195 and −497 [13]. Furthermore, the results indicated that deregulated miRNAs may contribute to posttranscriptional deregulation of signaling pathways which are known to be affected in meningiomas. While there are a number of already validated targets for miR-195 and miR-497, only a few are known for miR-136, −34a-3p and −376c [13].

For miR-34a-5p there are several studies showing a crucial role in the regulation of cell cycle, differentiation and apoptosis in different cancer types [14-16]. By contrast, the downstream effects of miR-34a-3p, which is downregulated in higher-grade meningiomas [13], are less well elucidated. It is, however, known that both the miR-34a-5p and the miR-34a-3p, which are derived from the pre-miR-34a, are active [17].

In the present study we focused on experimental validation of predicted targets of miR-34a-3p and the effects of deregulated miR-34a-3p in meningioma cells. In our *in silico* search we focused on putative targets that are components of cellular signaling pathways known to be affected in meningiomas. For experimental validation we selected Fos proto-oncogene, AP-1 transcription factor subunit (*FOS*) of the MAPK pathway [18], SMAD family member 4 (*SMAD4*) of the TGF-β pathway [19], frequently rearranged in advanced T-cell lymphomas 1 (*FRAT1*) of the Wnt/β-catenin pathway [20], SRC proto-oncogene, non-receptor tyrosine kinase (*SRC*) of the VEGF/PI3K pathway [21], and B-cell CLL/lymphoma 2 (*BCL2*) as a key regulator of apoptosis [22].

We also investigated the effects of overexpression or inhibition of miR-34a-3p on proliferation and apoptosis in meningioma cells *in vitro*.

## RESULTS

### *In silico* target search for miR-34a-3p

We previously showed that miR-34a-3p is significantly downregulated in WHO grade II and III meningiomas [13]. We performed a TargetScan Custom search with the seed sequence of miR-34a-3p and focused on predicted targets that are components of signaling pathways that are affected in meningiomas. Based on the results of this TargetScan Custom search, we selected the predicted targets *FOS*, *SMAD4*, *FRAT1*, *SRC* and *BCL2* for further experimental validation.

### Target validation for predicted targets of miR-34a-3p by dual luciferase assay

**Figure 1:**
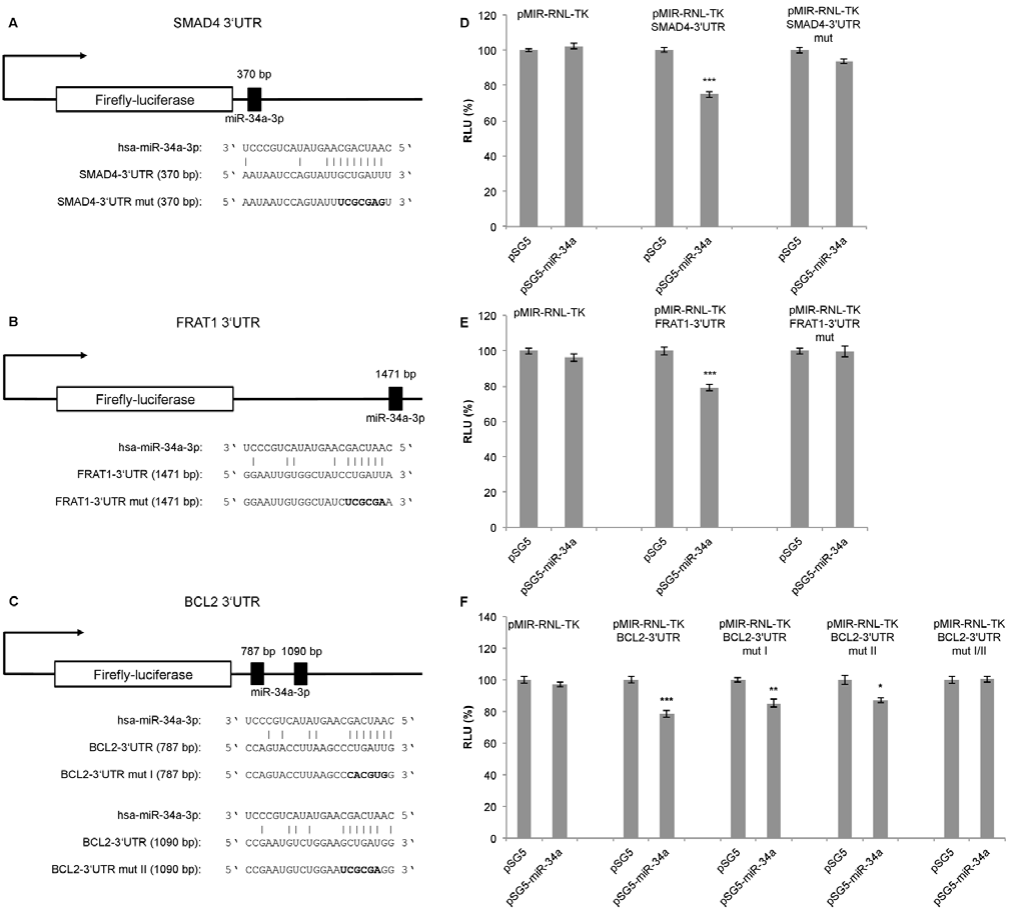
Dual luciferase reporter gene assays for *SMAD4*, *FRAT1* and *BCL2* (**A, B, C**) Schematic representation of luciferase reporter gene constructs with wild-type miRNA binding sites (3'UTR) and mutated variants (3'UTR mut) for *SMAD4*, *FRAT1* and *BCL2*. (**D**, **E**, **F**) Dual luciferase reporter gene assays 48 h after co-transfection of HEK293T cells with reporter gene constructs (pMIR-RNL-TK) with the respective wild-type or mutated 3'UTR for each target and control (pSG5) or miRNA-expression construct (pSG5-miR-34a) in the indicated combinations. Relative luciferase units (RLU) are means ± SEM of three independent experiments performed in duplicates (*, P < 0.05; **, P < 0.01; ***, P < 0.001).

To confirm the predicted targets of miR-34a-3p, we performed a dual luciferase assay using a pSG5-miR-34a expression vector. The miR-34a expression vector was co-expressed with the reporter gene vectors containing the 3'UTRs of the selected targets, each with the potential binding sites for miR-34a-3p. Following transfection of HEK293T cells with the pSG5-miR-34a expression vector, overexpression of miR-34a-3p was verified by quantitative Real-Time PCR (data not shown). Schematic representations of the reporter gene constructs with the predicted miR-34a-3p binding sites each for *SMAD4* and *FRAT1* are given in Fig. 1A and 1B. For *BCL2* we predicted two binding sites that are separated by approximately 300 bp as shown in Fig. 1C. Upon binding of miR-34a-3p to the *SMAD4* 3'UTR, the luciferase activity was reduced to 75 % (SEM ± 1.76, P < 0.001), and upon binding to the *FRAT1* 3'UTR the luciferase activity was reduced to 79 % (SEM ± 1.72, P < 0.001). After disruption of the potential binding sites the response to miR-34a-3p overexpression was lost (Fig. 1D and 1E). For the *BCL2* 3'UTR, which contains two potential binding sites, the luciferase activity was reduced to 78 % (SEM ± 2.36, P < 0.001). With one of the binding sites mutated, the luciferase activity was reduced to 85 % (SEM ± 2.36, P < 0.01) and with the other binding site mutated the luciferase activity was reduced to 87 % (SEM ± 1.67, P < 0.05). Upon disruption of both potential binding sites there was no responsiveness to miR-34a-3p overexpression (Fig. 1F). While the dual luciferase assays indicated a negative regulatory effect of miR-34a-3p on *SMAD4*, *FRAT1* and *BCL2* via binding to their 3'UTRs, we found no evidence for a regulatory effect of miR-34a-3p on the 3'UTRs of *FOS* and *SRC* (data not shown).

### Effect of ectopic deregulation of miR-34a-3p on protein expression of SMAD4, FRAT1 and BCL2

**Figure 2:**
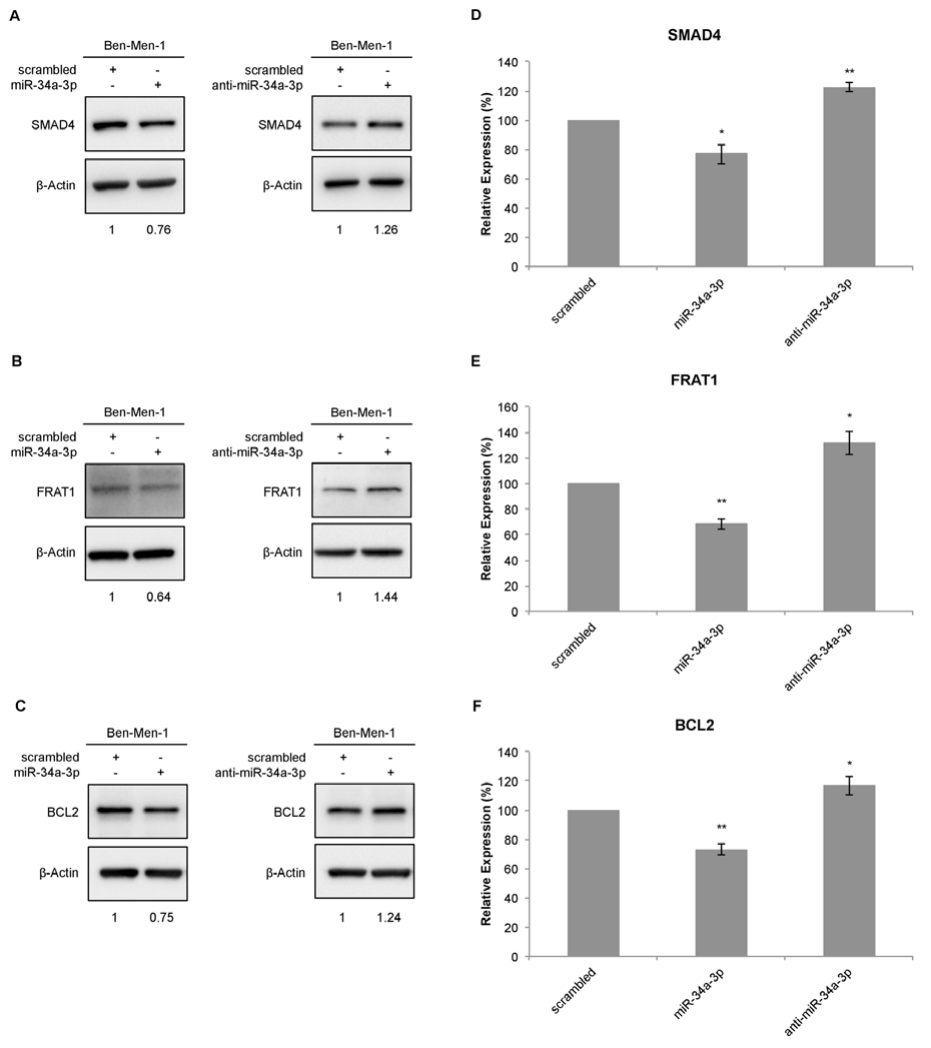
Western blot for SMAD4, FRAT1 and BCL2 protein expression after deregulation of miR-34a-3p in Ben-Men-1 cells (**A**, **B**, **C**) Representative Western blot images for SMAD4, FRAT1 and BCL2 protein expression 48 h after transfection of Ben-Men-1 cells with control (scrambled), miR-34a-3p (mimic) or anti-miR-34a-3p (inhibitor) using β-actin as loading control. (**D**, **E**, **F**) Quantification of SMAD4, FRAT1 and BCL2 protein expression relative to β-actin protein expression determined by Western blot. Values are means ± SD of three independent experiments (*, P < 0.05; **, P < 0.01).

To further determine the effects of miR-34a-3p on the selected targets, we analyzed the protein expression of the target genes in the cell line Ben-Men-1 that was derived from a meningothelial meningioma. Protein expression was analyzed by Western blotting 48 hours after transfection with miR-34a-3p or anti-miR-34a-3p. Scrambled miRNAs were used as control for off-target effects and β-actin as reference for quantification. Representative Western blots for SMAD4, FRAT1 and BCL2 are shown in Figure 2A-2C, respectively. After overexpression of miR-34a-3p, the protein level of SMAD4 was reduced to 76 % (SD ± 6.6, P < 0.05) and after inhibition of miR-34a-3p the protein level was increased to 122 % (SD ± 2.95, P < 0.01), each compared to scrambled controls (Fig. 2D). The FRAT1 protein level was reduced to 68 % (SD ± 4.1, P < 0.01) after overexpression of miR-34a-3p and increased to 131 % (SD ± 9.28, P < 0.05) after inhibition of miR-34a-3p (Fig. 2E). The BCL2 protein level was reduced to 73 % (SD ± 3.75, P < 0.01) after overexpression of miR-34a-3p and increased to 116% (SD ± 6.32, P < 0.05) after inhibition of miR-34a-3p, again relative to the scrambled controls (Fig. 2F).

### Overexpression of miR-34a-3p reduces the proliferation of meningioma cells

**Figure 3:**
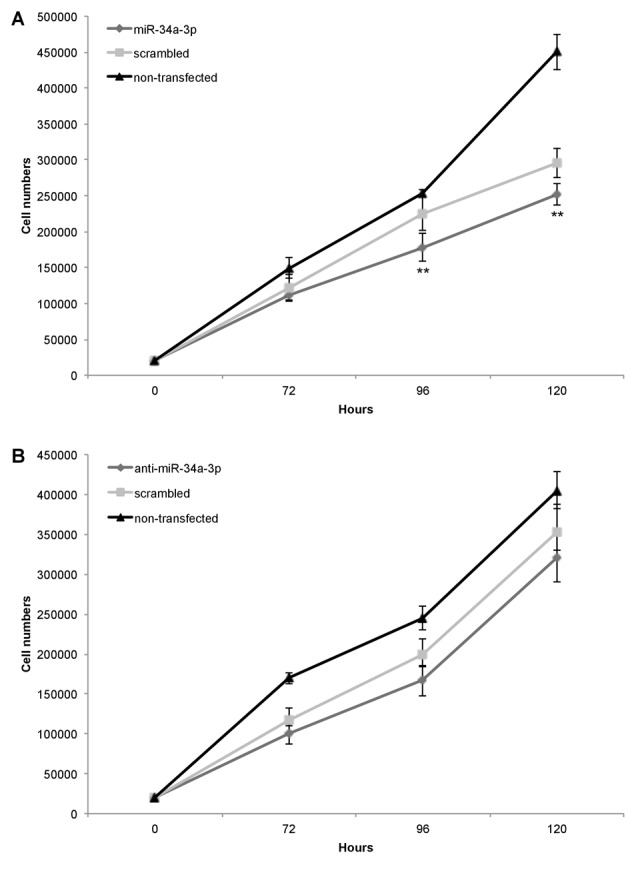
Overexpression of miR-34a-3p decreases meningioma cell proliferation Ben-Men-1 cells were transfected with either miR-34a-3p (mimic), scrambled control or left untransfected (**A**) or anti-miR-34a-3p (inhibitor), scrambled control or left untransfected (**B**). Cells were harvested and counted at indicated time points after transfection. Proliferation assays were performed in triplicate. Values are means ± SD (**, P < 0.01). Total cell counts are given in Supplemental Tables S1 and S2.

Our previous study showed that slow growing WHO grade I meningiomas have an overall higher expression level of miR-34a-3p than WHO grade II and III meningiomas [13]. Based on these findings, we chose the benign WHO grade I meningioma cell line Ben-Men-1 to analyze the effects of deregulation of miR-34a-3p on the process of malignant transformation of meningiomas. Since two of the experimentally validated targets, SMAD4 and FRAT1, are components of signaling pathways associated with activation of cellular proliferation, we tested the effects of miR-34a-3p overexpression or inhibition on the proliferation of Ben-Men-1 cells. Cells were transfected with miR-34a-3p or anti-miR-34a-3p, and counted each 72, 96 or 120 hours following transfection. As controls we used cells transfected with scrambled miRNAs and untransfected cells. Overexpression of miR-34a-3p decreased proliferation of Ben-Men-1 cells moderate but significant at the 96 and 120 hours time-point, respectively (Fig. 3A). However, inhibition of miR-34a-3p did not increase proliferation of Ben-Men-1 cells (Fig. 3B). Our findings support the idea that increased miR-34a-3p levels may contribute to a decreased cellular proliferation of meningioma cells *in vitro* via reduced SMAD4 and FRAT1 levels.

### Apoptosis is significantly decreased after inhibition of miR-34a-3p

**Figure 4:**
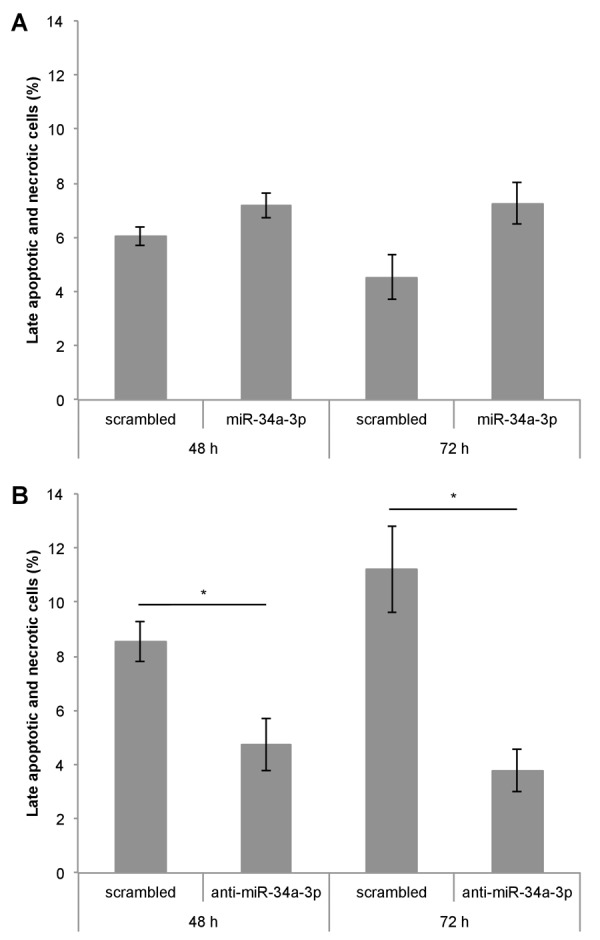
Deregulation of miR-34a-3p alters apoptosis and necrosis of meningioma cells Ben-Men-1 cells were transfected either with miR-34a-3p (mimic) and scrambled control (**A**) or anti-miR-34a-3p (inhibitor) and scrambled control (**B**). Cells were harvested 48 h and 72 h after transfection, stained with Annexin V / PI and analyzed by FACS. Values are means ± SEM of three independent experiments (*, P < 0.05).

One of our experimentally validated targets of miR-34a-3p is the anti-apoptotic protein BCL2. To examine the effects of altered miR-34a-3p levels on apoptosis, we transfected Ben-Men-1 cells with miR-34a-3p or anti-miR-34a-3p. As controls we used scrambled miRNAs. The apoptosis was measured by FACS with Annexin V / PI staining at two time points i.e. 48 and 72 hours after transfection. Overexpression of miR-34a-3p resulted in a distinct but not significantly increased amount of late apoptotic and necrotic cells compared to the scrambled control (Fig. 4A). Transfection with anti-miR-34a-3p led to a significant decrease in amount of late apoptotic and necrotic cells both at 48 and 72 hours as compared to the scrambled control (Fig. 4B). There was also a significant difference in the direct comparison after overexpression or inhibition of miR-34a-3p after 72 h (7.3 % vs. 3.8 %, P < 0.05). Our data indicate that a low miR-34a-3p level seems to be associated with a lower apoptotic rate of meningioma cells *in vitro*.

### Immunohistochemical detection of FRAT1 and SMAD4 expression in meningioma tumor tissue

We performed immunohistochemistry for detection of FRAT1 and SMAD4 protein levels in FFPE samples of 35 patients of our previous study [13], including 20 WHO grade I, 10 WHO grade II and 5 WHO grade III meningiomas, and correlated Immuno-Reactive-Score (IRS) with Ki-67 labeling index as measure of cell proliferation. Representative images can be found in Supplemental Figure S1 and IRS for all samples in Supplemental Table S5. For both proteins, there was a tendency toward higher expression in tumors with higher Ki-67 labeling index, but only in the group of tumors with Ki-67 ≥10.

## DISCUSSION

Previously, we reported downregulation of miR-34a-3p in higher-grade meningiomas [13]. To decipher the downstream effects of deregulation of miR-34a-3p, we performed an *in silico* analysis for identification of predicted targets of miR-34a-3p. Subsequently, we validated *SMAD4*, *FRAT1* and *BCL2* as direct targets of miR-34a-3p by dual luciferase assays and Western blotting. These targets are components of signaling pathways that are affected during meningioma genesis and progression [9, 10].

Alterations of these signaling pathways are associated with aberrant cell proliferation and apoptosis and therefore we tested the influence of overexpression or inhibition of miR-34a-3p on meningioma cells *in vitro*. We found that deregulation of miR-34a-3p affects cell proliferation and apoptosis of Ben-Men-1 cells.

**Figure 5:**
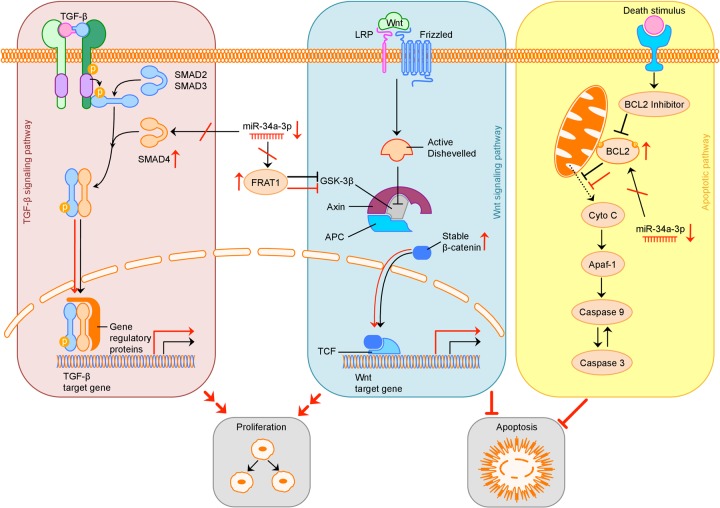
Overview of possible influence of downregulation of miR-34a-3p on the TGF-β, Wnt and apoptotic signaling pathway Activation (sharp arrows) or inhibition (blunt arrows) in normal signaling is indicated in black. Aberrant activation or inhibition, downregulation of miR-34a-3p and upregulation of target proteins is indicated in red. Deregulation of TGF-β pathway by overexpression of SMAD4 may increase cell proliferation, deregulation of Wnt pathway by overexpression of FRAT1 may increase cell proliferation and inhibit apoptosis. Overexpression of BCL2 may inhibit apoptosis. TGF-β: transforming growth factor β; LRP: low-density lipoprotein receptor-related protein; GSK-3β: glycogen synthase kinase 3β; APC: adenomatous polyposis coli; TCF: T-cell factor; Cyto C: Cytochrome C; Apaf-1: apoptotic peptidase activating factor 1.

The possible consequences of downregulation of miR-34a-3p and therefore loss of translational repression of *SMAD4*, *FRAT1* and *BCL2* on the TGF-β, Wnt and apoptotic signaling pathway are summarized in Figure 5.

SMAD4 is a major component of the TGF-β signaling pathway [19]. TGF-β signaling plays a dual role in tumor pathogenesis [19]. In early stages of tumor formation it functions as tumor suppressor by inhibiting cell proliferation and inducing apoptosis, whereas in later stages of tumor pathogenesis it induces EMT, angiogenesis and suppression of the immune system [19].

Mutations or alterations of the *SMAD4* gene, which maps to chromosome 18q, have been observed for several cancer types, including colon, lung and pancreas [23-25]. Although loss of chromosome 18q occurs in higher-grade meningiomas, alterations of the *SMAD4* gene locus apparently do not play a role in the pathogenesis of meningiomas [26].

Activation of TGF-β signaling induces EMT leading to downregulation of epithelial markers like E-cadherin and upregulation of mesenchymal markers like vimentin and N-cadherin [27, 28]. In meningiomas, E-cadherin expression is negatively correlated with WHO grade and invasion [29], while vimentin is increased with higher malignancy grades [30]. Downregulation of miR-200a, which was found in benign meningiomas compared to arachnoidal tissue, leads to an increased protein level of its direct targets i.e. the transcriptional repressors ZEB1 (zinc finger E-box binding homeobox 1) and SIP1/ZEB2 (zinc finger E-box binding homeobox 2), which in turn decreases transcription of E-cadherin, thereby promoting tumor growth and invasion [11]. These observations show that EMT is crucial for the progression towards higher-grade meningiomas [31].

*SMAD4* is also a direct target of miR-34a-5p in cholangiocarcinoma (CC) [32]. SMAD4 was associated with EMT as upregulation of miR-34a-5p and therefore downregulation of SMAD4 in CC cell lines repressed EMT via the TGF-β signaling pathway [32]. Recently, it was shown that miR-144 suppresses cell proliferation, migration and invasion in hepatocellular carcinoma (HCC) by directly targeting SMAD4 [33].

Taken together, these studies support the idea that downregulation of miR-34a-3p, especially in higher-grade meningiomas, may contribute to EMT, increased cell proliferation, invasion and migration of tumor cells via loss of translational repression of *SMAD4*. This scenario is consistent with our result that shows decreased meningioma cell proliferation *in vitro* as result of overexpression of miR-34a-3p.

To date, there is no knowledge about the role of FRAT1 in meningiomas. FRAT proteins are potent activators of the Wnt signaling pathway [20]. FRAT binds to glycogen synthase kinase 3β (GSK3β) and thereby inhibits the phosphorylation and subsequent degradation of β-catenin, resulting in increased translocation of β-catenin to the nucleus and transcriptional activation of downstream target genes of the β-catenin/T-cell factor (TCF) complex [20]. In esophageal squamous cell carcinoma (ESCC), overexpression of FRAT1 is associated with increased cell proliferation and aberrant activation of the β-catenin/TCF pathway, a consequence of nuclear accumulation of β-catenin that promotes the transcriptional activity of β-catenin/TCF [34]. Interestingly, also higher-grade meningiomas exhibit increased protein level and nuclear/perinuclear localization of β-catenin [31, 35], suggesting aberrant activation of the Wnt signaling pathway [9]. In gliomas, overexpression of FRAT1 is correlated with a malignant phenotype, higher cell proliferation, decreased apoptosis and poor prognosis [36, 37]. In non-small cell lung cancer (NSCLC) expression of FRAT1 correlates with β-catenin expression and is associated with tumor differentiation, tumor stage and lymph node metastasis [38, 39]. Knockdown of *FRAT1* expression by RNA interference resulted in inhibition of cell proliferation, migration and invasion of human glioblastoma cells [40] and also inhibition of proliferation and increased apoptosis of human gastric adenocarcinoma cells [41].

FRAT1 overexpression leads to increased proliferation and reduced apoptosis in several tumors [36, 37, 40, 41]. This observation is consistent with our result that downregulation of miR-34a-3p reduced apoptosis of meningioma cells *in vitro*. The exhibited nuclear accumulation of β-catenin in higher-grade meningiomas [31, 35] may be, in part, due to loss of translational repression of *FRAT1* due to the decreased levels of miR-34a-3p.

The role of BCL2 as key regulator of apoptosis is well investigated and documented [22]. There are a few studies on the anti-apoptotic role of BCL2 in meningiomas [42-44]. Higher expression of BCL2 was correlated with recurrence of benign meningiomas [43] and a shorter time-to-recurrence for patients with atypical meningiomas [42]. Meningiomas with complete or partial monosomy 22 showed an increased BCL2 protein level [44]. BCL2 also regulates migration, invasion and metastasis in several cancers including gliomas [45, 46], squamous cell carcinoma [47], neuroblastoma [48], lung [49] and colorectal cancer [50].

*BCL2* is a target of several miRNAs including miR-136 [51], miR-195 [52] and miR-497 [53], all of which were downregulated in higher-grade meningiomas as shown in our previous study [13]. In addition, similar to SMAD4, *BCL2* is also a validated target of miR-34a-5p [54]. Restoration of miR-34a-5p, which was downregulated in hepatocellular carcinoma (HCC), reduced cancer cell viability and promoted apoptosis [54].

Since higher-grade meningiomas showed downregulation of miR-34a-3p and other miRNAs [13] that directly target *BCL2*, we propose a crucial role for BCL2 overexpression in meningiomas. The overexpression of BCL2 is possibly associated with reduced apoptosis, invasion and migration, particularly in higher-grade meningiomas. This is consistent with our result that downregulation of miR-34a-3p reduced apoptosis of meningioma cells *in vitro*.

Taken together, we were able to validate three new direct targets for miR-34a-3p, SMAD4, FRAT1 and BCL2, which are likely to be implicated in the deregulation of signaling pathways that are already known to be involved in meningioma genesis and progression. We observed moderate but significant effects on meningioma cell proliferation and apoptosis in Ben-Men-1 cells. The hypothesized effects of downregulation of SMAD4 and FRAT1 on proliferation and upregulation of BCL2 on apoptosis of meningioma cells and the link to deregulation of miR-34a-3p should be explored further, also in malignant meningioma cell lines. In addition, further experiments are needed to elucidate the whole regulatory pattern of miR-34a-3p in signaling pathways in meningiomas *in vivo*.

## MATERIALS AND METHODS

### Cell culture

Human benign meningioma cell line Ben-Men-1 and HEK293T were purchased from DSMZ (Deutsche Sammlung von Mikroorganismen und Zellkulturen, Braunschweig, Germany). Ben-Men-1 was originally established from a meningothelial meningioma after surgical tumor resection [55]. The cells were immortalized by retroviral transduction with human telomerase reverse transcriptase (hTERT) gene [55].

Ben-Men-1 and HEK293T cells were cultured in Dulbecco's Modified Eagle Medium (Life Technologies, Carlsbad, CA, USA) supplemented with 10 % (v/v) heat-inactivated fetal bovine serum (FBS; Biochrom, Berlin, Germany) and 1 % (w/v) penicillin/streptomycin (P/S; Life Technologies, Carlsbad, CA, USA) at 37 °C and 5 % CO2.

### miRNA target screening

We performed a TargetScan Custom [56] search using the seed sequence of miR-34a-3p to identify predicted targets. For further validation experiments, we focused on targets that are associated with signaling pathways that are affected in meningiomas or crucial for proliferation, invasion, migration, EMT and apoptosis.

### miRNA overexpression and inhibition

Deregulation of miR-34a-3p expression in Ben-Men-1 cells was performed using miR-34a-3p (mimic) or anti-miR-34a-3p (inhibitor). AllStars Negative Control siRNA and miScript Inhibitor Negative Control were used as scrambled RNA negative controls for mimic and inhibitor, respectively. Transfections were performed using the lipid-based HiPerFect transfection reagent according to manufacturer's instructions. Transfection conditions were optimized using AllStars Cell Death Control siRNA (all from Qiagen, Hilden, Germany).

### Cell proliferation assay

Ben-Men-1 cells were seeded in 6-well plates with a starting amount of 20000 cells and transfected the same day with either miR-34a-3p (mimic, 10 nM final concentration) or anti-miR-34a-3p (inhibitor, 100 nM final concentration), and respective scrambled RNA negative controls in same final concentration or left untransfected. The transfection was performed in triplicate, medium was refreshed five hours after transfection. Cells were harvested by trypsinization and counted in duplicates per well 72, 96 and 120 hours post-transfection with an automated cell counter (Luna^™^ *fl*; Logos Biosystems, Annandale, VA, USA).

### Fluorescence-activated cell sorting analysis

Fluorescence-activated cell sorting (FACS) analysis was performed to determine the effects of miR-34a-3p overexpression or inhibition on apoptosis/necrosis of Ben-Men-1 cells. Cells (1 x 10^5^) were seeded in 6-well plates and transfected the same day with miR-34a-3p (mimic, 10 nM final concentration) or anti-miR-34a-3p (inhibitor, 100 nM final concentration). Same concentrations were used for the respective scrambled RNA negative controls.

Apoptosis/necrosis detection was performed with the Dead cell apoptosis kit with Alexa Fluor^®^ 488 Annexin V and Propidium Iodide (PI) for flow cytometry (Life Technologies, Carlsbad, CA, USA). Medium of each well was collected 48 or 72 hours after transfection, cells were harvested by trypsinization and added to the collected medium. Cells were pelleted at 300 x g for 3 min, cell pellets were washed with PBS, resuspended in 1x annexin binding buffer and 100 μl of the cell suspension were stained with 1 μl Alexa Fluor^®^ 488 Annexin V and 1 μl PI (100 μg/ml). After 15 min incubation, FACS measurement was performed on FACScan flow cytometer (BD Biosciences, Franklin Lakes, New Jersey, USA). A total of 10000 or 20000 cells per sample were analyzed for the 48 and 72 hours time point, respectively. Cells that stained positive for Annexin V, that binds to phosphatidylserine residues on the outer cell membrane, were defined as early apoptotic, whereas cells that stained positive for Annexin V and PI were defined as late apoptotic or necrotic, as described elsewhere [57].

### Plasmids

The pSG5 expression construct for miR-34a-5p and miR-34a-3p was cloned by *de novo* synthesis of the sequence for the miR-34a-5p/-3p precursor (nucleotides 9151617-9151816 on chromosome 1 (hg19)) and ligation of this fragment into pSG5 (Agilent Technologies, Santa Clara, CA, USA) using *Eco*RI and *Bgl*II restriction sites by Eurofins Genomics (Ebersberg, Germany).

A 792 bp fragment of the *SMAD4* 3'UTR (NM_005359.5, nucleotides 2199-2990) was PCR-amplified from cDNA using primers 5'-SMAD4-SpeI and 3'-SMAD4-SacI and cloned into the pMIR-RNL-TK dual-luciferase reporter gene vector. pMIR-RNL-TK is a derivate of pMIR-REPORT (Ambion/Thermo Fisher Scientific, Waltham, MA, USA) which is modified as described previously [58]. The cloned fragment incorporates one predicted binding site for miR-34a-3p and brings the firefly luciferase gene under the regulatory control of the *SMAD4* 3'UTR.

The potential binding site for miR-34a-3p was mutated in an overlap extension PCR. In detail, the specific primers for the *SMAD4* 3'UTR were used in combination with a mutagenesis primer overlapping the miRNA binding site and replacing it with an enzyme restriction site. Using pMIR-RNL-TK *SMAD4*-3'UTR as template a construct with a mutated miRNA binding site for miR-34a-3p was cloned.

A 469 bp fragment of the *FRAT1* 3'UTR (NM_005479.3, nucleotides 2157-2625) was PCR amplified, cloned and the miR-34a-3p binding site was mutated as described above.

A 716 bp fragment of the *BCL2* 3'UTR (NM_000633.2, nucleotides 1796-2511) was *de novo* synthesized and cloned into the pEX-A2 plasmid by Eurofins Genomics (Ebersberg, Germany). Cloning into pMIR-RNL-TK was performed via *Spe*I and *NgoM*IV restriction sites that were added during gene synthesis.

The two potential binding sites for miR-34a-3p in the *BCL2* 3'UTR were mutated as described above. Specific primers for pMIR-RNL-TK were used in combination with mutagenesis primers. Using pMIR-RNL-TK *BCL2*-3'UTR as template, constructs with one or both miRNA binding sites mutated, were cloned.

For sequences of all primers used for amplification and mutagenesis of 3'UTR fragments see Supplemental Table S3.

Since the miRNA expression plasmid expresses miR-34a-5p and miR-34a-3p all 3'UTR fragments for predicted targets were cloned excluding any potential binding sites for miR-34a-5p.

### Transfection and dual luciferase assays

HEK293T cells (0,4 x 10^5^) were seeded in 24-well plates and transfected the following day with 0,2 μg reporter gene construct and 0,8 μg miRNA precursor expression plasmid per well using PolyFect transfection reagent (Qiagen, Hilden, Germany) according to manufacturer's recommendations. Dual luciferase assays were performed 48 h after transfection using the Dual-Luciferase® Reporter Assay System (Promega, Mannheim, Germany) according to manufacturer's instructions.

### Western blot

Western blotting was performed to determine the effects of miR-34a-3p overexpression or inhibition on the predicted targets on protein level. Ben-Men-1 cells (1,2 x 10^5^) were seeded in 6-well plates and transfected the same day with miR-34a-3p (mimic, 10 nM final concentration) or anti-miR-34a-3p (inhibitor, 100 nM final concentration). Same concentrations were used for the related scrambled RNA negative controls. Cells were harvested 48 hours after transfection and cell pellets were lysed with 2x sample buffer (130 mM Tris/HCl, 6 % SDS, 10 % 3-mercapto-1,2-propandiol, 10 % glycerol). Fifteen μg of extracted proteins were separated by 12 % SDS-PAGE and transferred to a PVDF membrane (*Hybond^™^-P*, GE Healthcare, Little Chalfont, GB) by Western blotting. Primary antibodies included anti-SMAD4, anti-BCL2 (#9515 and #2876; Cell Signaling Technology, Danvers, MA, USA) and anti-FRAT1 (ab108405, Abcam, Cambridge, UK) at a final dilution of 1:1000. Primary anti-β-actin antibody (AC-15; Sigma, St. Louis, MO, USA) was used in a final dilution of 1:5000. Appropriate secondary antibodies (Dianova, Hamburg, Germany) were used in a final dilution of 1:1000 (FRAT1), 1:2500 (SMAD4 and BCL2) or 1:7500 (β-actin). Bands were visualized by enhanced chemiluminescence with SignalFire™ ECL Reagent (Cell Signaling Technology, Danvers, MA, USA). Detection was performed with the ChemiDoc™ Touch Imaging System and band intensities were quantified using β-actin as reference with the Image Lab™ software (Bio-Rad, Hercules, CA, USA).

### Patient samples

For immunohistochemistry protein expression, FFPE samples from 35 patients with meningiomas of different WHO grades and histological subtypes were selected retrospectively (for clinical details, see Supplemental Table S4). Patients were informed consent and tumor samples were obtained during surgery. After reclassification and confirmation of representativeness of WHO grading and histological subtypes by a neuropathologist, FFPE samples were used for immunohistochemical staining.

### Immunohistochemistry

Immunohistochemical stains of FFPE tumor samples were done using a BenchMark ULTRA automated immunostainer (Ventana Medical Systems, Inc., Tucson, AZ, USA).

Immunohistochemical expression of SMAD4 (monoclonal mouse anti-human antibody, MA5-15682, Invitrogen, Carlsbad, CA, USA) and FRAT1 (polyclonal rabbit anti-human antibody, PA5-29145, Invitrogen, Carlsbad, CA, USA) was semiquantified by using the Immuno-Reactive-Score (IRS) [59]. The IRS is the product of the number of the percentage of positively labelled cells (0: 0 %, 1: < 10 %, 2: 10-50 %, 3: 50-80 %, 4: >80 %) and the staining intensity (0: no staining, 1: mild, 2: moderate, 3: strong), which results in scores between 0 and 12.

### Data analysis and statistical methods

Statistical analyses were performed using Excel (Microsoft, Redmond, WA, USA). Two-sided unpaired Student's *t*-tests were performed for the proliferation, apoptosis and dual luciferase experiments. A one-sided unpaired Student's *t*-test was performed for quantification of relative up- or downregulation of proteins in the Western blotting experiments. P-values < 0.05 were considered significant.

## SUPPLEMENTARY MATERIALS FIGURES AND TABLES


