# Supplementary material for: MiR-34a-3p alters proliferation and apoptosis of meningioma cells in vitro and is directly targeting SMAD4, FRAT1 and BCL2
Source: Aging (Albany NY). 2017 Mar 23;9(3):932–44. doi: 10.18632/aging.101201 (PMC5391240; doi:10.18632/aging.101201)
Supplement: Supplementary file 1 [file aging-09-932-s001.pdf]

SUPPLEMENTARY MATERIALS

Supplemental Figure.

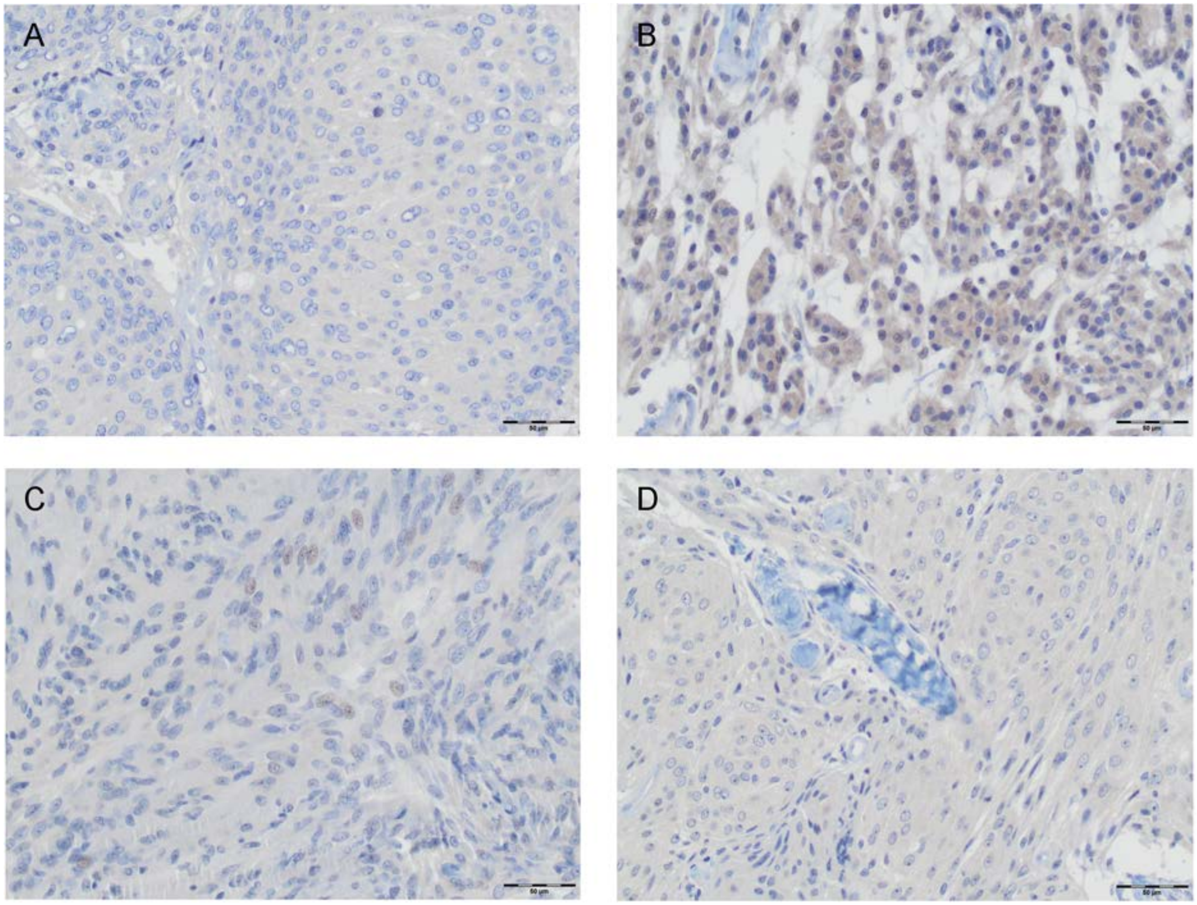

Supplemental Figure S1. Immunohistochemistry for detection of FRAT1 and SMAD4 protein levels in FFPE samples. Protein levels were semiquantified using the Immuno-Reactive-Score (IRS). (**A, B**) Representative images for FRAT1 staining for samples with an IRS of 4 (**A**) and 12 (**B**). (**C, D**) Representative images for SMAD4 staining with an IRS of 4 (**C**) and 8 (**D**). Scale bar 50 µm.

Supplemental Tables.

Table S1. Raw cell counts for Ben-Men-1 cells after transfection with miR-34a-3p.

	miR-34a-3p					scrambled					non-transfected				
Hours	1	2	3	Mean	SD	1	2	3	Mean	SD	1	2	3	Mean	SD
0	20000	20000	20000	20000	0	20000	20000	20000	20000	0	20000	20000	20000	20000	0
	20000	20000	20000			20000	20000	20000			20000	20000	20000		
72	117000	106000	124000	112000	7724,42	143000	113000	147000	121883,33	18735,03	154000	145000	176000	149500	14705,44
	117000	104000	104000			104000	97300	127000			143000	127000	152000		
96	179000	156000	152000	178000	19874,61	244000	199000	251000	225000	23194,83	251000	256000	263000	253166,67	5112,62
	204000	201000	176000			224000	242000	190000			251000	251000	247000		
120	258000	267000	235000	251666,67	14510,53	285000	315000	269000	295000	20289,57	468000	471000	414000	450333,33	25031,09
	256000	265000	229000			292000	281000	328000			453000	419000	477000		

Table S2. Raw cell counts for Ben-Men-1 cells after transfection with anti-miR-34a-3p.

	anti-miR-34a-3p					scrambled					non-transfected				
Hours	1	2	3	Mean	SD	1	2	3	Mean	SD	1	2	3	Mean	SD
0	20000	20000	20000	20000	0	20000	20000	20000	20000	0	20000	20000	20000	20000	0
	20000	20000	20000			20000	20000	20000			20000	20000	20000		
72	104000	88200	102000	99866,67	12623,61	106000	131000	106000	117216,67	14484,52	174000	172000	174000	169666,67	7063,21
	124000	95000	86000			129000	97300	134000			172000	154000	172000		
96	140000	163000	183000	166666,67	19618,59	231000	213000	181000	199166,67	19878,10	251000	226000	274000	245333,33	14985,18
	170000	147000	197000			174000	208000	188000			244000	242000	235000		
120	274000	367000	344000	320166,67	30218,19	403000	364000	315000	353000	34539,83	432000	434000	382000	405166,67	22843,06
	296000	321000	319000			337000	387000	312000			403000	407000	373000		

Table S3. Primer sequences for plasmid cloning and mutagenesis.

Name	Sequence
pMIR-RNL-TK for	GAAGTACCGAAAGGTCTTACCG
pMIR-RNL-TK rev	CCAAGCTAGCGGCCGCATACAA
pMIR-BCL2 Mut BS1 for	CCAGTACCTTAAGCC**CACGTG**GTGTATATTCATATATTTTG
pMIR-BCL2 Mut BS1 rev	CAAAATATATGAATATACAC**CACGTG**GGCTTAAGGTACTGG
pMIR-BCL2 Mut BS2 for	CTCCGAATGTCTGGAA**TCGCGA**GGAGCTCAGAATTCCAC
pMIR-BCL2 Mut BS2 rev	GTGGAATTCTGAGCTCC**TCGCGA**TTCCAGACATTCGGAG
5'-SMAD4-SpeI	GGACTAGTGTCTTTTACCGTTGGGG
3'-SMAD4-SacI	CGAGCTCCCAACCTTGTGCCTAG
pMIR-SMAD4 Mut for	GAATAATCCAGTATT**TCGCGAG**TTAAAGGCAGAGAAG
pMIR-SMAD4 Mut rev	CTTCTCTGCCTTTAA**CTCGCGA**AATACTGGATTATTC
5'-FRAT1-SpeI	GGACTAGTGCACAGCAGCTTATAATGG
3'-FRAT1-SacI	CGAGCTCGGAGATCAGAGAAATGTG
pMIR-FRAT1 Mut for	GGAATTGTGGCTATC**TCGCGA**ATAGGATTTTAACTTAAC
pMIR-FRAT1 Mut rev	GTTAAGTTAAAATCCTAT**TCGCGA**GATAGCCACAATTCC

Restriction sites are underlined, mutated sites are indicated in bold

Table S4. Clinical data on patients for immunohistochemistry.

ID	Age	Sex	WHO grading	Histological subtype	Ki-67 labeling index
2358	63,95	f	I	meningothelial	4
2364	56,49	m	I	meningothelial	2
2368	62,79	m	I	microcystic	<1
2382	79,20	m	I	meningothelial	1
2401	58,08	f	I	meningothelial	3
2415	65,88	f	I	fibroblastic	2
2431	47,24	f	I	meningothelial	<5
2437	62,43	f	I	transitional	1
2443	46,59	f	I	fibroblastic	8
2442	61,95	m	I	angiomatous	4
2464	70,87	m	I	meningothelial	3
2463	63,15	m	I	meningothelial	1
2466	73,02	f	I	meningothelial	3
2498	81,58	f	I	meningothelial	3
2503	33,01	f	I	fibroblastic	5
2505	47,56	f	I	meningothelial	2
2523	59,57	f	I	secretory	<1
2549	46,80	f	I	transitional	5
2558	51,22	f	I	fibroblastic	2
2564	72,60	f	I	transitional	N/A
2371	39,30	f	II	atypical	15
2378	70,48	m	II	brain invasive	3
2385	86,61	f	II	atypical	12
2395	50,70	m	II	atypical	20
2399	68,10	f	II	atypical	10
2400	13,33	m	II	atypical	20
2408	52,16	m	II	chordoid	20
2423	80,42	f	II	atypical	10
2426	70,21	f	II	brain invasive	10
2427	46,92	m	II	atypical	5
2547	60,44	f	III	anaplastic	20
2553	87,52	f	III	anaplastic	20
2581	87,71	m	III	anaplastic	20
2594	74,12	m	III	anaplastic	50
2410	75,33	m	III	anaplastic	30

Table S5. Results of immunohistochemistry.

					FRAT1	SMAD4
**ID**	**Age**	**Sex**	**WHO grading**	**Histological subtype**	**IRS**	**IRS**
2358	63,95	f	I	meningothelial	12	4
2364	56,49	m	I	meningothelial	12	8
2368	62,79	m	I	microcystic	12	6
2382	79,20	m	I	meningothelial	4	4
2401	58,08	f	I	meningothelial	8	8
2415	65,88	f	I	fibroblastic	4	2
2431	47,24	f	I	meningothelial	8	0
2437	62,43	f	I	transitional	8	4
2443	46,59	f	I	fibroblastic	3	0
2442	61,95	m	I	angiomatous	4	3
2464	70,87	m	I	meningothelial	4	0
2463	63,15	m	I	meningothelial	4	0
2466	73,02	f	I	meningothelial	8	4
2498	81,58	f	I	meningothelial	8	3
2503	33,01	f	I	fibroblastic	4	0
2505	47,56	f	I	meningothelial	4	8
2523	59,57	f	I	secretory	4	0
2549	46,80	f	I	transitional	1	3
2558	51,22	f	I	fibroblastic	0	0
2564	72,60	f	I	transitional	8	3
2371	39,30	f	II	atypical	4	0
2378	70,48	m	II	brain invasive	3	6
2385	86,61	f	II	atypical	4	2
2395	50,70	m	II	atypical	4	3
2399	68,10	f	II	atypical	4	0
2400	13,33	m	II	atypical	8	8
2408	52,16	m	II	chordoid	12	0
2423	80,42	f	II	atypical	2	1
2426	70,21	f	II	brain invasive	4	4
2427	46,92	m	II	atypical	8	4
2547	60,44	f	III	anaplastic	0	3
2553	87,52	f	III	anaplastic	3	3
2581	87,71	m	III	anaplastic	8	4
2594	74,12	m	III	anaplastic	6	3
2410	75,33	m	III	anaplastic	8	3

Immuno-Reactive-Score (IRS) for FRAT1 and SMAD4 immunostaining

mRNA sequences of targets for experimental validation

1. Homo sapiens SMAD family member 4 (SMAD4), mRNA

The stop codon is highlighted in red, the potential binding site for miR-34a-3p is highlighted in green and the complete fragment cloned into pMIR-RNL-TK is underlined.

NCBI Reference Sequence: NM_005359.5

```
>gi|195963400|ref|NM_005359.5| Homo sapiens SMAD family member 4 (SMAD4), mRNA

ATGCTCAGTGGCTTCTCGACAAGTTGGCAGCAACAACACGGCCCTGGTCGTCGCCGCTGCGGTAACGGAGCGGTTTGGGTGG
CGGAGCCTGCGTTCGCGCCTTCCCGCTCTCCTCGGGAGGCCCTTCCTGCTCTCCCCTAGGCTCCGCGGCCGCCCAGGGGGTGGGA
GCGGGTGAGGGGAGCCAGGCGCCCAGCGAGAGAGGCCCCCCGCCGCAGGGCGGCCCGGGAGCTCGAGGCGGTCCGGCCCGCGCGG
GCAGCGGCGCGGCGCTGAGGAGGGGCGGCCTGGCCGGGACGCCTCGGGGCGGGGGCCGAGGAGCTCTCCGGGCCGCCGGGGAAAG
CTACGGGCCCGGTGCGTCCGCGGACCAGCAGCGCGGGAGAGCGGACTCCCCTCGCCACCGCCCGAGCCCAGGTTATCCTGAATAC
ATGTCTAACAATTTTCCTTGCAACGTTAGCTGTTGTTTTTCACTGTTTCCAAAGGATCAAAATTGCTTCAGAAATTGGAGACATA
TTTGATTTAAAAGGAAAAACTTGAACAAATGGACAATATGTCTATTACGAATACACCAACAAGTAATGATGCCTGTCTGAGCATT
GTGCATAGTTTGATGTGCCATAGACAAGGTGGAGAGAGTGAAACATTTGCAAAAAGAGCAATTGAAAGTTTGGTAAAGAAGCTGA
AGGAGAAAAAAGATGAATTGGATTCTTTAATAACAGCTATAACTACAAATGGAGCTCATCCTAGTAAATGTGTTACCATACAGAG
AACATTGGATGGGAGGCTTCAGGTGGCTGGTCGGAAAGGATTTCCTCATGTGATCTATGCCCGTCTCTGGAGGTGGCCTGATCTT
CACAAAAATGAACTAAAACATGTTAAATATTGTCAGTATGCGTTTGACTTAAAATGTGATAGTGTCTGTGTGAATCCATATCACT
ACGAACGAGTTGTATCACCTGGAATTGATCTCTCAGGATTAACACTGCAGAGTAATGCTCCATCAAGTATGATGGTGAAGGATGA
ATATGTGCATGACTTTGAGGGACAGCCATCGTTGTCCACTGAAGGACATTCAATTCAAACCATCCAGCATCCACCAAGTAATCGT
GCATCGACAGAGACATACAGCACCCCAGCTCTGTTAGCCCCATCTGAGTCTAATGCTACCAGCACTGCCAACTTTCCCAACATTC
CTGTGGCTTCCACAAGTCAGCCTGCCAGTATACTGGGGGGCAGCCATAGTGAAGGACTGTTGCAGATAGCATCAGGGCCTCAGCC
AGGACAGCAGCAGAATGGATTTACTGGTCAGCCAGCTACTTACCATCATAACAGCACTACCACCTGGACTGGAAGTAGGACTGCA
CCATACACACCTAATTTGCCTCACCACCAAAACGGCCATCTTCAGCACCACCCGCCTATGCCGCCCCATCCCGGACATTACTGGC
CTGTTCACAATGAGCTTGCATTCCAGCCTCCCATTTCCAATCATCCTGCTCCTGAGTATTGGTGTTCCATTGCTTACTTTGAAAT
GGATGTTCAGGTAGGAGAGACATTTAAGGTTCCTTCAAGCTGCCCTATTGTTACTGTTGATGGATACGTGGACCCTTCTGGAGGA
GATCGCTTTTGTTTGGGTCAACTCTCCAATGTCCACAGGACAGAAGCCATTGAGAGAGCAAGGTTGCACATAGGCAAAGGTGTGC
AGTTGGAATGTAAAGGTGAAGGTGATGTTTGGGTCAGGTGCCTTAGTGACCACGCGGTCTTTGTACAGAGTTACTACTTAGACAG
AGAAGCTGGGCGTGCACCTGGAGATGCTGTTCATAAGATCTACCCAAGTGCATATATAAAGGTCTTTGATTTGCGTCAGTGTCAT
CGACAGATGCAGCAGCAGGCGGCTACTGCACAAGCTGCAGCAGCTGCCCAGGCAGCAGCCGTGGCAGGAAACATCCCTGGCCCAG
GATCAGTAGGTGGAATAGCTCCAGCTATCAGTCTGTCAGCTGCTGCTGGAATTGGTGTTGATGACCTTCGTCGCTTATGCATACT
CAGGATGAGTTTTGTGAAAGGCTGGGGACCGGATTACCCAAGACAGAGCATCAAAGAAACACCTTGCTGGATTGAAATTCACTTA
CACCGGGCCCTCCAGCTCCTAGACGAAGTACTTCATACCATGCCGATTGCAGACCCACAACCTTTAGACTGAGGTCTTTTACCGT
TGGGGCCCTTAACCTTATCAGGATGGTGGACTACAAAATACAATCCTGTTTATAATCTGAAGATATATTTCACTTTTGTTCTGCT
TTATCTTTTCATAAAGGGTTGAAAATGTGTTTGCTGCCTTGCTCCTAGCAGACAGAAACTGGATTAAAACAATTTTTTTTTTCCT
CTTCAGAACTTGTCAGGCATGGCTCAGAGCTTGAAGATTAGGAGAAACACATTCTTATTAATTCTTCACCTGTTATGTATGAAGG
AATCATTCCAGTGCTAGAAAATTTAGCCCTTTAAAACGTCTTAGAGCCTTTTATCTGCAGAACATCGATATGTATATCATTCTAC
AGAATAATCCAGTATTGCTGATTTTAAAGGCAGAGAAGTTCTCAAAGTTAATTCACCTATGTTATTTTGTGTACAAGTTGTTATT
GTTGAACATACTTCAAAAATAATGTGCCATGTGGGTGAGTTAATTTTACCAAGAGTAACTTTACTCTGTGTTTAAAAAGTAAGTT
AATAATGTATTGTAATCTTTCATCCAAAATATTTTTTGCAAGTTATATTAGTGAAGATGGTTTCAATTCAGATTGTCTTGCAACT
TCAGTTTTATTTTTGCCAAGGCAAAAAACTCTTAATCTGTGTGTATATTGAGAATCCCTTAAAATTACCAGACAAAAAAATTTAA
AATTACGTTTGTTATTCCTAGTGGATGACTGTTGATGAAGTATACTTTTCCCCTGTTAAACAGTAGTTGTATTCTTCTGTATTTC
TAGGCACAAGGTTGGTTGCTAAGAAGCCTATAAGAGGAATTTCTTTTCCTTCATTCATAGGGAAAGGTTTTGTATTTTTTAAAAC
ACTAAAAGCAGCGTCACTCTACCTAATGTCTCACTGTTCTGCAAAGGTGGCAATGCTTAAACTAAATAATGAATAAACTGAATAT
TTTGGAAACTGCTAAATTCTATGTTAAATACTGTGCAGAATAATGGAAACATTACAGTTCATAATAGGTAGTTTGGATATTTTTG
TACTTGATTTGATGTGACTTTTTTTGGTATAATGTTTAAATCATGTATGTTATGATATTGTTTAAAATTCAGTTTTTGTATCTTG
GGGCAAGACTGCAAACTTTTTTATATCTTTTGGTTATTCTAAGCCCTTTGCCATCAATGATCATATCAATTGGCAGTGACTTTGT
ATAGAGAATTTAAGTAGAAAAGTTGCAGATGTATTGACTGTACCACAGACACAATATGTATGCTTTTTACCTAGCTGGTAGCATA
AATAAAACTGAATCTCAACATACAAAGTTGAATTCTAGGTTTGATTTTTAAGATTTTTTTTTTCTTTTGCACTTTTGAGTCCAAT
CTCAGTGATGAGGTACCTTCTACTAAATGACAGGCAACAGCCAGTTCTATTGGGCAGCTTTGTTTTTTTCCCTCACACTCTACCG
GGACTTCCCCATGGACATTGTGTATCATGTGTAGAGTTGGTTTTTTTTTTTTTTTAATTTTTATTTTACTATAGCAGAAATAGACC
TGATTATCTACAAGATGATAAATAGATTGTCTACAGGATAAATAGTATGAAATAAAATCAAGGATTATCTTTCAGATGTGTTTAC
TTTTGCCTGGAGAACTTTTAGCTATAGAAACACTTGTGTGATGATAGTCCTCCTTATATCACCTGGAATGAACACAGCTTCTACT
GCCTTGCTCAGAAGGTCTTTTAAATAGACCATCCTAGAAACCACTGAGTTTGCTTATTTCTGTGATTTAAACATAGATCTTGATC
```

CAAGCTACATGACTTTTGTCTTTAAATAACTTATCTACCACCTCATTTGTACTCTTGATTACTTACAAATTCTTTCAGTAAACAC
CTAATTTTCTTCTGTAAAAGTTTGGTGATTTAAGTTTTATTGGCAGTTTTATAAAAAGACATCTTCTCTAGAAATTGCTAACTTT
AGGTCCATTTTACTGTGAATGAGGAATAGGAGTGAGTTTTAGAATAACAGATTTTTAAAAATCCAGATGATTTGATTAAAACCTT
AATCATACATTGACATAATTCATTGCTTCTTTTTTTTGAGATATGGAGTCTTGCTGTGTTGCCCAGGCAGGAGTGCAGTGGTATG
ATCTCAGCTCACTGCAACCTCTGCCTCCCGGGTTCAACTGATTCTCCTGCCTCAGCCTCCCTGGTAGCTAGGATTACAGGTGCCC
GCCACCATGCCTGGCTAACTTTTGTAGTTTTAGTAGAGACGGGGTTTTGCCTGTTGGCCAGGCTGGTCTTGAACTCCTGACCTCA
AGTGATCCATCCACCTTGGCCTCCCAAAGTGCTGGGATTACGGGCGTGAGCCACTGTCCCTGGCCTCATTGTTCCCTTTTCTACT
TTAAGGAAAGTTTTCATGTTTAATCATCTGGGGAAAGTATGTGAAAAATATTTGTTAAGAAGTATCTCTTTGGAGCCAAGCCACC
TGTCTTGGTTTCTTTCTACTAAGAGCCATAAAGTATAGAAATACTTCTAGTTGTTAAGTGCTTATATTTGTACCTAGATTTAGTC
ACACGCTTTTGAGAAAACATCTAGTATGTTATGATCAGCTATTCCTGAGAGCTTGGTTGTTAATCTATATTTCTATTTCTTAGTG
GTAGTCATCTTTGATGAATAAGACTAAAGATTCTCACAGGTTTAAAATTTTATGTCTACTTTAAGGGTAAAATTATGAGGTTATG
GTTCTGGGTGGGTTTTCTCTAGCTAATTCATATCTCAAAGAGTCTCAAAATGTTGAATTTCAGTGCAAGCTGAATGAGAGATGAG
CCATGTACACCCACCGTAAGACCTCATTCCATGTTTGTCCAGTGCCTTTCAGTGCATTATCAAAGGGAATCCTTCATGGTGTTGC
CTTTATTTTCCGGGGAGTAGATCGTGGGATATAGTCTATCTCATTTTTAATAGTTTACCGCCCCTGGTATACAAAGATAATGACA
ATAAATCACTGCCATATAACCTTGCTTTTTCCAGAAACATGGCTGTTTTGTATTGCTGTAACCACTAAATAGGTTGCCTATACCA
TTCCTCCTGTGAACAGTGCAGATTTACAGGTTGCATGGTCTGGCTTAAGGAGAGCCATACTTGAGACATGTGAGTAAACTGAACT
CATATTAGCTGTGCTGCATTTCAGACTTAAAATCCATTTTTGTGGGGCAGGGTGTGGTGTGTAAAGGGGGGTGTTTGTAATACAA
GTTGAAGGCAAAATAAAATGTCCTGTCTCCCAGATGATATACATCTTATTATTTTTAAAGTTTATTGCTAATTGTAGGAAGGTGA
GTTGCAGGTATCTTTGACTATGGTCATCTGGGGAAGGAAAATTTTACATTTTACTATTAATGCTCCTTAAGTGTCTATGGAGGTT
AAAGAATAAAATGGTAAATGTTTCTGTGCCTGGTTTGATGGTAACTGGTTAATAGTTACTCACCATTTTATGCAGAGTCACATTA
GTTCACACCCTTTCTGAGAGCCTTTTGGGAGAAGCAGTTTTATTCTCTGAGTGGAACAGAGTTCTTTTTGTTGATAATTTCTAGT
TTGCTCCCTTCGTTATTGCCAACTTTACTGGCATTTTATTTAATGATAGCAGATTGGGAAAATGGCAAATTTAGGTTACGGAGGT
AAATGAGTATATGAAAGCAATTACCTCTAAAGCCAGTTAACAATTATTTTGTAGGTGGGGTACACTCAGCTTAAAGTAATGCATT
TTTTTTTCCCGTAAAGGCAGAATCCATCTTGTTGCAGATAGCTATCTAAATAATCTCATATCCTCTTTTGCAAAGACTACAGAGA
ATAGGCTATGACAATCTTGTTCAAGCCTTTCCATTTTTTTCCCTGATAACTAAGTAATTTCTTTGAACATACCAAGAAGTATGTA
AAAAGTCCATGGCCTTATTCATCCACAAAGTGGCATCCTAGGCCCAGCCTTATCCCTAGCAGTTGTCCCAGTGCTGCTAGGTTGC
TTATCTTGTTTATCTGGAATCACTGTGGAGTGAAATTTTCCACATCATCCAGAATTGCCTTATTTAAGAAGTAAAACGTTTTAAT
TTTTAGCCTTTTTTTGGTGGAGTTATTTAATATGTATATCAGAGGATATACTAGATGGTAACATTTCTTTCTGTGCTTGGCTATC
TTTGTGGACTTCAGGGGCTTCTAAAACAGACAGGACTGTGTTGCCTTTACTAAATGGTCTGAGACAGCTATGGTTTTGAATTTTT
AGTTTTTTTTTTTTAACCCACTTCCCCTCCTGGTCTCTTCCCTCTCTGATAATTACCATTCATATGTGAGTGTTAGTGTGCCTCC
TTTTAGCATTTTCTTCTTCTCTTTCTGATTCTTCATTTCTGACTGCCTAGGCAAGGAAACCAGATAACCAAACTTACTAGAACGT
TCTTTAAAACACAAGTACAAACTCTGGGACAGGACCCAAGACACTTTCCTGTGAAGTGCTGAAAAAGACCTCATTGTATTGGCAT
TTGATATCAGTTTGATGTAGCTTAGAGTGCTTCCTGATTCTTGCTGAGTTTCAGGTAGTTGAGATAGAGAGAAGTGAGTCATATT
CATATTTTCCCCCTTAGAATAATATTTTGAAAGGTTTCATTGCTTCCACTTGAATGCTGCTCTTACAAAAACTGGGGTTACAAGG
GTTACTAAATTAGCATCAGTAGCCAGAGGCAATACCGTTGTCTGGAGGACACCAGCAAACAACACACAACAAAGCAAAACAAACC
TTGGGAAACTAAGGCCATTTGTTTTGTTTTGGTGTCCCCTTTGAAGCCCTGCCTTCTGGCCTTACTCCTGTACAGATATTTTTGA
CCTATAGGTGCCTTTATGAGAATTGAGGGTCTGACATCCTGCCCCAAGGAGTAGCTAAAGTAATTGCTAGTGTTTTCAGGGATTT
TAACATCAGACTGGAATGAATGAATGAAACTTTTTGTCCTTTTTTTTTCTGTTTTTTTTTTTCTAATGTAGTAAGGACTAAGGAA
AACCTTTGGTGAAGACAATCATTTCTCTCTGTTGATGTGGATACTTTTCACACCGTTTATTTAAATGCTTTCTCAATAGGTCCAG
AGCCAGTGTTCTTGTTCAACCTGAAAGTAATGGCTCTGGGTTGGGCCAGACAGTTGCACTCTCTAGTTTGCCCTCTGCCACAAAT
TTGATGTGTGACCTTTGGGCAAGTCATTTATCTTCTCTGGGCCTTAGTTGCCTCATCTGTAAAATGAGGGAGTTGGAGTAGATTA
ATTATTCCAGCTCTGAAATTCTAAGTGACCTTGGCTACCTTGCAGCAGTTTTGGATTTCTTCCTTATCTTTGTTCTGCTGTTTGA
GGGGGCTTTTTACTTATTTCCATGTTATTCAAAGGAGACTAGGCTTGATATTTTATTACTGTTCTTTTATGGACAAAAGGTTACA
TAGTATGCCCTTAAGACTTAATTTTAACCAAAGGCCTAGCACCACCTTAGGGGCTGCAATAAACACTTAACGCGCGTGCGCACGC
GCGCGCGCACACACACACACACACACACACACACACACAGGTCAGAGTTTAAGGCTTTCGAGTCATGACATTCTAGCTTTTGAAT
TGCGTGCACACACACACGCACGCACACACTCTGGTCAGAGTTTATTAAGGCTTTCGAGTCATGACATTATAGCTTTTGAGTTGGT
GTGTGTGACACCACCCTCCTAAGTGGTGTGTGCTTGTAATTTTTTTTTTCAGTGAAAATGGATTGAAAACCTGTTGTTAATGCTT
AGTGATATTATGCTCAAAACAAGGAAATTCCCTTGAACCGTGTCAATTAAACTGGTTTATATGACTCAAGAAAACAATACCAGTA
GATGATTATTAACTTTATTCTTGGCTCTTTTTAGGTCCATTTTGATTAAGTGACTTTTGGCTGGATCATTCAGAGCTCTCTTCTA
GCCTACCCTTGGATGAGTACAATTAATGAAATTCATATTTTCAAGGACCTGGGAGCCTTCCTTGGGGCTGGGTTGAGGGTGGGGG
GTTGGGGAGTCCTGGTAGAGGCCAGCTTTGTGGTAGCTGGAGAGGAAGGGATGAAACCAGCTGCTGTTGCAAAGGCTGCTTGTCA
TTGATAGAAGGACTCACGGGCTTGGATTGATTAAGACTAAACATGGAGTTGGCAAACTTTCTTCAAGTATTGAGTTCTGTTCAAT
GCATTGGACATGTGATTTAAGGGAAAAGTGTGAATGCTTATAGATGATGAAAACCTGGTGGGCTGCAGAGCCCAGTTTAGAAGAA
GTGAGTTGGGGGTTGGGGACAGATTTGGTGGTGGTATTTCCCAACTGTTTCCTCCCCTAAATTCAGAGGAATGCAGCTATGCCAG
AAGCCAGAGAAGAGCCACTCGTAGCTTCTGCTTTGGGGACAACTGGTCAGTTGAAAGTCCCAGGAGTTCCTTTGTGGCTTTCTGT
ATACTTTTGCCTGGTTAAAGTCTGTGGCTAAAAAATAGTCGAACCTTTCTTGAGAACTCTGTAACAAAGTATGTTTTTGATTAAA
AGAGAAAGCCAACTAAAAAAAAAAAAAAAAAAAA

2. Homo sapiens frequently rearranged in advanced T-cell lymphomas 1 (FRAT1), mRNA

The stop codon is highlighted in red, the potential binding site for miR-34a-3p is highlighted in green and the complete fragment cloned into pMIR-RNL-TK is underlined.

NCBI Reference Sequence: NM_005479.3

```
>gi|197313686|ref|NM_005479.3| Homo sapiens frequently rearranged in advanced T-cell
lymphomas 1 (FRAT1), mRNA

GGATTCCGGCTCCCGCGGCTGCAGGCGCGGCTAGAGTGCCTGGCGGGCTCCGGCTTCCGCGTCCGCCCCGGCCCCGGTCCAGA
CTTAGTCTTCAGCTCCGCGCCCGCTCCGCCGCGGCCCACCGCGCCCGCCGGCAGCCGAGCCCCCAGCGACGCCCGCACAGCTCCG
GGTGCCCAGACAGGGGGCCATGCCGTGCCGGAGGGAGGAGGAAGAGGAAGCCGGCGAGGAGGCGGAGGGGGAGGAAGAGGAGGAG
GACAGCTTCCTCCTACTGCAGCAGTCAGTGGCGCTGGGCAGCTCGGGCGAGGTGGACCGGCTGGTGGCCCAGATCGGCGAGACGC
TGCAGCTGGACGCGGCGCAGCACAGCCCGGCCTCGCCGTGCGGGCCCCCGGGGGCGCCGCTGCGGGCCCCGGGGCCCCTGGCTGC
GGCGGTGCCGGCGGACAAGGCCAGGTCCCCGGCGGTGCCGCTGCTGCTGCCGCCCGCGTTGGCGGAGACTGTGGGCCCGGCGCCC
CCTGGGGTCCTGCGCTGCGCCCTGGGGGACCGCGGCCGCGTGCGGGGCCGCGCTGCGCCCTACTGCGTGGCCGAGCTCGCCACAG
GCCCCAGCGCGCTGTCCCCACTGCCCCCTCAGGCCGACCTTGATGGGCCTCCGGGAGCTGGCAAGCAGGGCATCCCGCAGCCGCT
GTCGGGTCCGTGCCGGCGAGGATGGCTCCGGGGCGCCGCCGCCTCCCGCCGCCTGCAGCAGCGACGCGGGTCCCAACCAGAAACC
CGCACAGGCGACGACGACCCGCACCGGCTTCTGCAGCAGCTAGTGCTCTCTGGAAACCTCATCAAGGAGGCCGTGCGAAGGCTTC
ATTCGCGACGGCTGCAGTTACGTGCAAAGCTTCCCCAACGCCCGCTCCTGGGACCTCTGTCGGCCCCGGTGCATGAACCCCCTTC
GCCTCGCAGCCCTCGCGCGGCCTGCAGTGACCCTGGCGCCTCCGGGAGGGCGCAGCTCAGAACTGGCGACGGCGTTCTTGTGCCT
GGCAGCTAACACGCCCGGGGTGGCCACAGCGCCAGCCTCAGACTGGAGGGCAAGGGGTTCCCTTGAGGGCTGCAGTTCTACTCAG
GCTGGTGGAGAACTCTGGCTTTTGGAAGCGAGAGTAAAAAGCTAATGACGAGGAACCGAAAAATCGCGAGTGTTTCGCGGGTAAC
TGGGGTTGAGGGCCAAAATATTTGGAATGAAGGACTTTGGCCCTATTTAAGGCAGATTTTACAGAGCGCACCTCAAACGTACAAG
TCAGTAGGACTCCTTATTTGGCGTGACCCGACCTGGCCGCGGAGCCTGCATTTCCTCGCAGCCTCTCAGTGCCCTCCAGCCCCGC
GACCATGTGGCCACAATCCACGCTTCTCCGGATCGCGGTGCGCCGGAACCACGGAGGATGATGCCAGTTACTTGCTTTACCTTTT
CAGGGCTGGCTCCTGATCCACTTTGGGGGAGGAGAACATGAGTAGATAATTTCAGGGTGCAGCCCAATCTGCCAGACTTAAAAAA
ACCATCTTGTGTCTTTGGAGGTGCTGCTTAATACCAAACATGCGGTGCCATGAAGGGACCCTTTGGGGGTTGAATAGGAGTTAAC
CCCTGCGCTCTCTTTGCAACTGTCTCTCTTCTCAGAGTGGTGGGGGAAGGCTGTACGACACGGGTGGGGAAAGGAGGTGGGGGCG
GGGAGTATTGAATGGTGGTGGAAGGGTAGAGAGGCGCGGAGTGAACCCCACGCCCTGTCTAAAGTGTATTTTCAGAGCCGGCCCG
CCTCTCCTCGGTTCAAGGTCACTGTTTCCTGGGCACGCACTGGGTTGCGGGACAGAGTAGCCAGGTTCTGCCGGTGCTCGGAGAA
GAGCGCAGTGTTTTGCAAGTGCTGGAGTCTCCTGAGGACACGCGCGTCGCCGCCACCGCGGGTGTGGGAAAGCGCGGACGTGCTG
GGCGGCTGTGCTTCGGTAGGCGACCACCGCCCCTGGCCGCGCTCCGGGCTTTCACGGAAACTCCCGAGACCGGGCCCTGGGTTCC
TCCTCTCCTACTCGGCTCTGCAGTCCTACTCAAGCGGGTGGCTCTGGGATCCTGGGGGCCTGGGTTGGGGGCTAGGGAGACGCCA
TGTGATGGACACTCCAGGGACACACAGCCTAGCACAGCAGCTTATAATGGGCTCTCCGGGGCCATTTGCAATAACAGCTGCAATT
CCCTGGATAGACGAGTTGATTTCCTCCCTCTGCCCCTCCCCCAGCCATGCCAGCTGGCCTTTGTAAGTGCAGGAAACCGAGTAGA
AAATGTGACCCTCCAAATGGAGAAGCTGCAGGCTTTGCCATTGTGAACCATGGTGAAGTGCTTGGAACATACTGTTCACTCACTC
TAAAGGCGCTGAGACTGTGCTGTTGTTCTCGTTTTTATAGTCAATGGCTTGTTCATCATCCAGATGTGGCTACTGACATATCTAC
ACTTCGCACCGGAGTGTCTGGAATTGTGGCTATCCTGATTATAGGATTTTAACTTAACTGAAATGCCTGCTTTGAATAAATGTGT
TGGGTTTTTTGTTTGGTTTTATTTTATACTTGCCATCAGTGAAAAAGATGTACAGAACACATTTCTCTGATCTCCATAAACATGA
AAACACTTGAAATCTC
```

3. Homo sapiens B-cell CLL/lymphoma 2 (BCL2), transcript variant alpha, mRNA

The stop codon is highlighted in red, the potential binding sites for miR-34a-3p are highlighted in green and the complete fragment that was *de novo* synthesized and cloned into pMIR-RNL-TK is underlined

NCBI Reference Sequence: NM_000633.2

```
>gi|72198188|ref|NM_000633.2| Homo sapiens B-cell CLL/lymphoma 2 (BCL2), transcript
variant alpha, mRNA
TTTCTGTGAAGCAGAAGTCTGGGAATCGATCTGGAAATCCTCCTAATTTTTACTCCCTCTCCCCGCGACTCCTGATTCATTGGGA
AGTTTCAAATCAGCTATAACTGGAGAGTGCTGAAGATTGATGGGATCGTTGCCTTATGCATTTGTTTTGGTTTTACAAAAAGGAA
ACTTGACAGAGGATCATGCTGTACTTAAAAAATACAACATCACAGAGGAAGTAGACTGATATTAACAATACTTACTAATAATAAC
GTGCCTCATGAAATAAAGATCCGAAAGGAATTGGAATAAAAATTTCCTGCATCTCATGCCAAGGGGGAAACACCAGAATCAAGTG
TTCCGCGTGATTGAAGACACCCCCTCGTCCAAGAATGCAAAGCACATCCAATAAAATAGCTGGATTATAACTCCTCTTCTTTCTC
TGGGGGCCGTGGGGTGGGAGCTGGGGCGAGAGGTGCCGTTGGCCCCCGTTGCTTTTCCTCTGGGAAGGATGGCGCACGCTGGGAG
AACAGGGTACGATAACCGGGAGATAGTGATGAAGTACATCCATTATAAGCTGTCGCAGAGGGGCTACGAGTGGGATGCGGGAGAT
GTGGGCGCCGCGCCCCCGGGGGCCGCCCCCGCACCGGGCATCTTCTCCTCCCAGCCCGGGCACACGCCCCATCCAGCCGCATCCC
GGGACCCGGTCGCCAGGACCTCGCCGCTGCAGACCCCGGCTGCCCCCGGCGCCGCCGCGGGGCCTGCGCTCAGCCCGGTGCCACC
TGTGGTCCACCTGACCCTCCGCCAGGCCGGCGACGACTTCTCCCGCCGCTACCGCCGCGACTTCGCCGAGATGTCCAGCCAGCTG
CACCTGACGCCCTTCACCGCGCGGGGACGCTTTGCCACGGTGGTGGAGGAGCTCTTCAGGGACGGGGTGAACTGGGGGAGGATTG
TGGCCTTCTTTGAGTTCGGTGGGGTCATGTGTGTGGAGAGCGTCAACCGGGAGATGTCGCCCCTGGTGGACAACATCGCCCTGTG
GATGACTGAGTACCTGAACCGGCACCTGCACACCTGGATCCAGGATAACGGAGGCTGGGATGCCTTTGTGGAACTGTACGGCCCC
AGCATGCGGCCTCTGTTTGATTTCTCCTGGCTGTCTCTGAAGACTCTGCTCAGTTTGGCCCTGGTGGGAGCTTGCATCACCCTGG
GTGCCTATCTGGGCCACAAGTGAAGTCAACATGCCTGCCCCAAACAAATATGCAAAAGGTTCACTAAAGCAGTAGAAATAATATG
CATTGTCAGTGATGTACCATGAAACAAAGCTGCAGGCTGTTTAAGAAAAAATAACACACATATAAACATCACACACACAGACAGA
CACACACACACACAACAATTAACAGTCTTCAGGCAAAACGTCGAATCAGCTATTTACTGCCAAAGGGAAATATCATTTATTTTTT
ACATTATTAAGAAAAAAAGATTTATTTATTTAAGACAGTCCCATCAAAACTCCTGTCTTTGGAAATCCGACCACTAATTGCCAAG
CACCGCTTCGTGTGGCTCCACCTGGATGTTCTGTGCCTGTAAACATAGATTCGCTTTCCATGTTGTTGGCCGGATCACCATCTGA
AGAGCAGACGGATGGAAAAAGGACCTGATCATTGGGGAAGCTGGCTTTCTGGCTGCTGGAGGCTGGGGAGAAGGTGTTCATTCAC
TTGCATTTCTTTGCCCTGGGGGCTGTGATATTAACAGAGGGAGGGTTCCTGTGGGGGGAAGTCCATGCCTCCCTGGCCTGAAGAA
GAGACTCTTTGCATATGACTCACATGATGCATACCTGGTGGGAGGAAAAGAGTTGGGAACTTCAGATGGACCTAGTACCCACTGA
GATTTCCACGCCGAAGGACAGCGATGGGAAAAATGCCCTTAAATCATAGGAAAGTATTTTTTTAAGCTACCAATTGTGCCGAGAA
AAGCATTTTAGCAATTTATACAATATCATCCAGTACCTTAAGCCCTGATTGTGTATATTCATATATTTTGGATACGCACCCCCCA
ACTCCCAATACTGGCTCTGTCTGAGTAAGAAACAGAATCCTCTGGAACTTGAGGAAGTGAACATTTCGGTGACTTCCGCATCAGG
AAGGCTAGAGTTACCCAGAGCATCAGGCCGCCACAAGTGCCTGCTTTTAGGAGACCGAAGTCCGCAGAACCTGCCTGTGTCCCAG
CTTGGAGGCCTGGTCCTGGAACTGAGCCGGGGCCCTCACTGGCCTCCTCCAGGGATGATCAACAGGGCAGTGTGGTCTCCGAATG
TCTGGAAGCTGATGGAGCTCAGAATTCCACTGTCAAGAAAGAGCAGTAGAGGGGTGTGGCTGGGCCTGTCACCCTGGGGCCCTCC
AGGTAGGCCCGTTTTCACGTGGAGCATGGGAGCCACGACCCTTCTTAAGACATGTATCACTGTAGAGGGAAGGAACAGAGGCCCT
GGGCCCTTCCTATCAGAAGGACATGGTGAAGGCTGGGAACGTGAGGAGAGGCAATGGCCACGGCCCATTTTGGCTGTAGCACATG
GCACGTTGGCTGTGTGGCCTTGGCCCACCTGTGAGTTTAAAGCAAGGCTTTAAATGACTTTGGAGAGGGTCACAAATCCTAAAAG
AAGCATTGAAGTGAGGTGTCATGGATTAATTGACCCCTGTCTATGGAATTACATGTAAAACATTATCTTGTCACTGTAGTTTGGT
TTTATTTGAAAACCTGACAAAAAAAAAGTTCCAGGTGTGGAATATGGGGGTTATCTGTACATCCTGGGGCATTAAAAAAAAAATC
AATGGTGGGGAACTATAAAGAAGTAACAAAAGAAGTGACATCTTCAGCAAATAAACTAGGAAATTTTTTTTTCTTCCAGTTTAGA
ATCAGCCTTGAAACATTGATGGAATAACTCTGTGGCATTATTGCATTATATACCATTTATCTGTATTAACTTTGGAATGTACTCT
GTTCAATGTTTAATGCTGTGGTTGATATTTCGAAAGCTGCTTTAAAAAAATACATGCATCTCAGCGTTTTTTTGTTTTTAATTGT
ATTTAGTTATGGCCTATACACTATTTGTGAGCAAAGGTGATCGTTTTCTGTTTGAGATTTTTATCTCTTGATTCTTCAAAAGCAT
TCTGAGAAGGTGAGATAAGCCCTGAGTCTCAGCTACCTAAGAAAAACCTGGATGTCACTGGCCACTGAGGAGCTTTGTTTCAACC
AAGTCATGTGCATTTCCACGTCAACAGAATTGTTTATTGTGACAGTTATATCTGTTGTCCCTTTGACCTTGTTTCTTGAAGGTTT
CCTCGTCCCTGGGCAATTCCGCATTTAATTCATGGTATTCAGGATTACATGCATGTTTGGTTAAACCCATGAGATTCATTCAGTT
AAAAATCCAGATGGCAAATGACCAGCAGATTCAAATCTATGGTGGTTTGACCTTTAGAGAGTTGCTTTACGTGGCCTGTTTCAAC
ACAGACCCACCCAGAGCCCTCCTGCCCTCCTTCCGCGGGGGCTTTCTCATGGCTGTCCTTCAGGGTCTTCCTGAAATGCAGTGGT
GCTTACGCTCCACCAAGAAAGCAGGAAACCTGTGGTATGAAGCCAGACCTCCCCGGCGGGCCTCAGGGAACAGAATGATCAGACC
TTTGAATGATTCTAATTTTTAAGCAAAATATTATTTTATGAAAGGTTTACATTGTCAAAGTGATGAATATGGAATATCCAATCCT
GTGCTGCTATCCTGCCAAAATCATTTTAATGGAGTCAGTTTGCAGTATGCTCCACGTGGTAAGATCCTCCAAGCTGCTTTAGAAG
TAACAATGAAGAACGTGGACGTTTTTAATATAAAGCCTGTTTTGTCTTTTGTTGTTGTTCAAACGGGATTCACAGAGTATTTGAA
AAATGTATATATATTAAGAGGTCACGGGGGCTAATTGCTGGCTGGCTGCCTTTTGCTGTGGGGTTTTGTTACCTGGTTTTAATAA
CAGTAAATGTGCCCAGCCTCTTGGCCCCAGAACTGTACAGTATTGTGGCTGCACTTGCTCTAAGAGTAGTTGATGTTGCATTTTC
CTTATTGTTAAAAACATGTTAGAAGCAATGAATGTATATAAAAGCCTCAACTAGTCATTTTTTTCTCCTCTTCTTTTTTTTCATT
```

ATATCTAATTATTTTGCAGTTGGGCAACAGAGAACCATCCCTATTTTGTATTGAAGAGGGATTCACATCTGCATCTTAACTGCTC
TTTATGAATGAAAAAACAGTCCTCTGTATGTACTCCTCTTTACACTGGCCAGGGTCAGAGTTAAATAGAGTATATGCACTTTCCA
AATTGGGGACAAGGGCTCTAAAAAAAGCCCCAAAAGGAGAAGAACATCTGAGAACCTCCTCGGCCCTCCCAGTCCCTCGCTGCAC
AAATACTCCGCAAGAGAGGCCAGAATGACAGCTGACAGGGTCTATGGCCATCGGGTCGTCTCCGAAGATTTGGCAGGGGCAGAAA
ACTCTGGCAGGCTTAAGATTTGGAATAAAGTCACAGAATTAAGGAAGCACCTCAATTTAGTTCAAACAAGACGCCAACATTCTCT
CCACAGCTCACTTACCTCTCTGTGTTCAGATGTGGCCTTCCATTTATATGTGATCTTTGTTTTATTAGTAAATGCTTATCATCTA
AAGATGTAGCTCTGGCCCAGTGGGAAAAATTAGGAAGTGATTATAAATCGAGAGGAGTTATAATAATCAAGATTAAATGTAAATA
ATCAGGGCAATCCCAACACATGTCTAGCTTTCACCTCCAGGATCTATTGAGTGAACAGAATTGCAAATAGTCTCTATTTGTAATT
GAACTTATCCTAAAACAAATAGTTTATAAATGTGAACTTAAACTCTAATTAATTCCAACTGTACTTTTAAGGCAGTGGCTGTTTT
TAGACTTTCTTATCACTTATAGTTAGTAATGTACACCTACTCTATCAGAGAAAAACAGGAAAGGCTCGAAATACAAGCCATTCTA
AGGAAATTAGGGAGTCAGTTGAAATTCTATTCTGATCTTATTCTGTGGTGTCTTTTGCAGCCCAGACAAATGTGGTTACACACTT
TTTAAGAAATACAATTCTACATTGTCAAGCTTATGAAGGTTCCAATCAGATCTTTATTGTTATTCAATTTGGATCTTTCAGGGAT
TTTTTTTTTAAATTATTATGGGACAAAGGACATTTGTTGGAGGGGTGGGAGGGAGGAAGAATTTTTAAATGTAAAACATTCCCAA
GTTTGGATCAGGGAGTTGGAAGTTTTCAGAATAACCAGAACTAAGGGTATGAAGGACCTGTATTGGGGTCGATGTGATGCCTCTG
CGAAGAACCTTGTGTGACAAATGAGAAACATTTTGAAGTTTGTGGTACGACCTTTAGATTCCAGAGACATCAGCATGGCTCAAAG
TGCAGCTCCGTTTGGCAGTGCAATGGTATAAATTTCAAGCTGGATATGTCTAATGGGTATTTAAACAATAAATGTGCAGTTTTAA
CTAACAGGATATTTAATGACAACCTTCTGGTTGGTAGGGACATCTGTTTCTAAATGTTTATTATGTACAATACAGAAAAAAATTT
TATAAAATTAAGCAATGTGAAACTGAATTGGAGAGTGATAATACAAGTCCTTTAGTCTTACCCAGTGAATCATTCTGTTCCATGT
CTTTGGACAACCATGACCTTGGACAATCATGAAATATGCATCTCACTGGATGCAAAGAAAATCAGATGGAGCATGAATGGTACTG
TACCGGTTCATCTGGACTGCCCCAGAAAAATAACTTCAAGCAAACATCCTATCAACAACAAGGTTGTTCTGCATACCAAGCTGAG
CACAGAAGATGGGAACACTGGTGGAGGATGGAAAGGCTCGCTCAATCAAGAAAATTCTGAGACTATTAATAAATAAGACTGTAGT
GTAGATACTGAGTAAATCCATGCACCTAAACCTTTTGGAAAATCTGCCGTGGGCCCTCCAGATAGCTCATTTCATTAAGTTTTTC
CCTCCAAGGTAGAATTTGCAAGAGTGACAGTGGATTGCATTTCTTTTGGGGAAGCTTTCTTTTGGTGGTTTTGTTTATTATACCT
TCTTAAGTTTTCAACCAAGGTTTGCTTTTGTTTTGAGTTACTGGGGTTATTTTTGTTTTAAATAAAAATAAGTGTACAATAAGTG
TTTTTGTATTGAAAGCTTTTGTTATCAAGATTTTCATACTTTTACCTTCCATGGCTCTTTTTAAGATTGATACTTTTAAGAGGTG
GCTGATATTCTGCAACACTGTACACATAAAAAATACGGTAAGGATACTTTACATGGTTAAGGTAAAGTAAGTCTCCAGTTGGCCA
CCATTAGCTATAATGGCACTTTGTTTGTGTTGTTGGAAAAAGTCACATTGCCATTAAACTTTCCTTGTCTGTCTAGTTAATATTG
TGAAGAAAAATAAAGTACAGTGTGAGATACTG
